# Ube2W conjugates ubiquitin to α-amino groups of protein N-termini

**DOI:** 10.1042/BJ20130244

**Published:** 2013-06-13

**Authors:** Michael H. Tatham, Anna Plechanovová, Ellis G. Jaffray, Helena Salmen, Ronald T. Hay

**Affiliations:** Wellcome Trust Centre for Gene Regulation and Expression, Sir James Black Centre, College of Life Sciences, University of Dundee, Dow Street, Dundee DD1 5EH, U.K.

**Keywords:** E2 ubiquitin-conjugating enzyme, N-terminal modification, RNF4 (RING finger protein 4), SUMO (small ubiquitin-related modifier), ubiquitin, ubiquitin-conjugating enzyme E2W (Ube2W), CHIP, C-terminus of the Hsc (heat-shock cognate) 70-interacting protein, E1 enzyme, ubiquitin-activating enzyme, E2 enzyme, ubiquitin-conjugating enzyme, HCD, higher energy collisional dissociation, Isopep.6His-SUMO-2_×4_, isopeptide bond-linked His_6_-polySUMO-2 construct, Isopep.SUMO-2_×4_, isopeptide bond-linked polySUMO-2 construct, Pep.6His-SUMO-2_×4_, peptide bond-linked His_6_-polySUMO-2 construct, RNF4, RING finger protein 4, SENP1, sentrin-specific protease 1, SUMO, small ubiquitin-related modifier, TEV, tobacco etch virus, Ube2W, ubiquitin-conjugating enzyme E2W, UEV1, ubiquitin-conjugating enzyme E2 variant 1

## Abstract

The covalent attachment of the protein ubiquitin to intracellular proteins by a process known as ubiquitylation regulates almost all major cellular systems, predominantly by regulating protein turnover. Ubiquitylation requires the co-ordinated action of three enzymes termed E1, E2 and E3, and typically results in the formation of an isopeptide bond between the C-terminal carboxy group of ubiquitin and the ϵ-amino group of a target lysine residue. However, ubiquitin is also known to conjugate to the thiol of cysteine residue side chains and the α-amino group of protein N-termini, although the enzymes responsible for discrimination between different chemical groups have not been defined. In the present study, we show that Ube2W (Ubc16) is an E2 ubiquitin-conjugating enzyme with specific protein N-terminal mono-ubiquitylation activity. Ube2W conjugates ubiquitin not only to its own N-terminus, but also to that of the small ubiquitin-like modifier SUMO (small ubiquitin-related modifier) in a manner dependent on the SUMO-targeted ubiquitin ligase RNF4 (RING finger protein 4). Furthermore, N-terminal mono-ubiquitylation of SUMO-2 primes it for poly-ubiquitylation by the Ubc13–UEV1 (ubiquitin-conjugating enzyme E2 variant 1) heterodimer, showing that N-terminal ubiquitylation regulates protein fate. The description in the present study is the first of an E2-conjugating enzyme with N-terminal ubiquitylation activity, and highlights the importance of E2 enzymes in the ultimate outcome of E3-mediated ubiquitylation.

## INTRODUCTION

Ubiquitin is conjugated to target proteins in a three-step reaction where the ubiquitin is first adenylated, then covalently linked via a thioester bond to a cysteine residue in an E1 enzyme (ubiquitin-activating enzyme). The ubiquitin is then transferred via a second thioester bond on to an E2 enzyme (ubiquitin-conjugating enzyme). In human cells there are almost 40 different E2 enzymes that act as intermediates either transferring the ubiquitin on to an HECT (homologous with E6-associated protein C-terminus)-type E3 ubiquitin ligase or transferring the ubiquitin directly on to a substrate lysine residue with the help of a RING-type E3 [1]. The RING-containing SUMO (small ubiquitin-related modifier)-targeted ubiquitin E3 ligase RNF4 (RING finger protein 4) is responsible for the arsenic-induced ubiquitylation and degradation of the PML (promyelocytic leukaemia) protein [2,3]. RNF4 binds to poly-SUMO chains and partners with the UbcH4/5 family of E2 enzymes to generate poly-ubiquitin chains that target the modified protein for degradation [2]. Recently, it has been shown that RNF4 plays a role in the response of cells to DNA damage [4–7], specifically resulting in the accumulation of Lys^63^-linked poly-ubiquitin chains at damage sites. To determine whether other E2s could partner RNF4 and synthesize discrete Lys^63^ polymers on SUMOs, we screened a library of E2s and found that Ube2W (ubiquitin-conjugating enzyme E2W) could ubiquitylate poly-SUMO substrates in an RNF4-dependent manner. Surprisingly, MS analysis failed to detect substantial lysine residue modification, but did demonstrate quantitative modification of protein N-termini. Modification of SUMO chains on N-termini then rendered them amenable to RNF4-dependent ubiquitylation by Ubc13–UEV1 (ubiquitin-conjugating enzyme E2 variant 1), which synthesized Lys^63^-linked chains on to this newly primed substrate.

## EXPERIMENTAL

### *In vitro* ubiquitin conjugation assays

Unless otherwise stated, *in vitro* ubiquitin conjugation reactions were conducted in 10–50 μl volumes containing 50 mM Tris, pH 7.5, 3 mM ATP, 5 mM MgCl_2_, 150 mM NaCl, 0.5 mM TCEP [tris-(2-carboxyethyl)phosphine] and 0.1% Nonidet P40. Various combinations of protein constituents were used at the following concentrations: 1–2.5 μM E2 enzyme, 20 μM ubiquitin, 35–100 nM His-tagged UBE1, 5.5 μM substrate, 0.55 μM RNF4 and 1 μM CHIP [C-terminus of the Hsc (heat-shock cognate) 70-interacting protein]. Reactions were incubated at 20 or 37°C for the times indicated in the Figure legends.

### Identification of ubiquitylation sites by MS

For in-solution analysis, *in vitro* conjugation reactions were halted by the addition of SDS to 2% and Tris/HCl (pH 7.6) to 50 mM, then essentially the FASP protocol [8] was followed. For in-gel analyses, reaction products from *in vitro* ubiquitin conjugation assays, halted by the addition of SDS sample buffer containing reducing agent, were fractionated by SDS/PAGE (Novex NuPAGE 10% Bis-Tris gel; Life Technologies) using Mes SDS running buffer. Tryptic peptides were generated by an in-gel digestion method [9]. For partial digestion analyses, trypsin was used at 1 ng·μl^−1^. Alkylation was induced by chloroacetamide. Between 0.5 and 1 μg of each peptide sample was analysed by MS using a Q Exactive LC–MS/MS (liquid chromatography–tandem MS) mass spectrometer (Thermo Fisher Scientific) coupled with a Proxeon nano-HPLC system (Proxeon Biosystems). Typically peptides were analysed using a 60-min elution gradient from 5 to 80% acetonitrile, fractionating on a 150-mm reverse-phase C_18_, 3 μm, 100 Å (1 Å=0.1 nm) and 75 μm ID column (Acclaim PepMap 100; Thermo Scientific) coupled with a nanoelectrospray ion source. To detect ubiquitin-SUMO-2 peptides, gradients were modified to begin at 0% acetonitrile. MS data were acquired using a data-dependent top-10 method dynamically choosing the most abundant precursor ions from the survey scan for HCD (higher energy collisional dissociation). Survey scans (*m*/*z* 300–1700) were acquired at a resolution of 70000 at *m*/*z* 200 (after accumulation to a target value of 1000000). Resolution for HCD spectra was set to 17500 at *m*/*z* 200.

Raw MS data files were processed by MaxQuant (version 1.3.0.5) [10,11]. Data were searched against an entire human proteome database plus one containing the known recombinant proteins in the reactions either with or without an N-terminal extension of LRGG to represent N-terminal ubiquitylation. Importantly, two forms of each protein were included, either containing or omitting the N-terminal methionine residue. Variable modifications were methionine oxidation, protein N-acetylation, and GG and LRGG lysine adducts. The minimum peptide length was set to five amino acids and a maximum of three or four missed cleavages depending on whether partial digestion was employed. A 1% FDR (false discovery rate) was required at both the protein and peptide level. Spectra were annotated using the MaxQuant spectral annotation system as well as the ‘Expert system’ [12].

### cDNA cloning

Human Ube2W (NCBI NP_001001481.1; now updated to NP_001001481.2) was subcloned into pHIS-TEV-30a vector using NcoI and HindIII restriction sites. As a result of cloning, the construct contains five extra N-terminal residues (GAMGS) once cleaved with TEV (tobacco etch virus) protease.

### Recombinant protein expression

The sequences of all proteins used in the biochemical assays described in the present study can be found in the Supplementary Online Data (at http://www.biochemj.org/bj/453/bj4530137add.htm). Ubiquitin from bovine erythrocytes was commercially sourced (Sigma–Aldrich). Most proteins were expressed as His_6_- or His_6_-maltose binding protein-tagged proteins in bacteria and purified by nickel-affinity chromatography. In most cases, tags were removed by TEV protease digestion, followed by repurification of the untagged product. This strategy has been described previously [2].

## RESULTS

### Ube2W conjugates ubiquitin to SUMO-2 polymers in an RNF4-dependent manner

**Figure 1:**
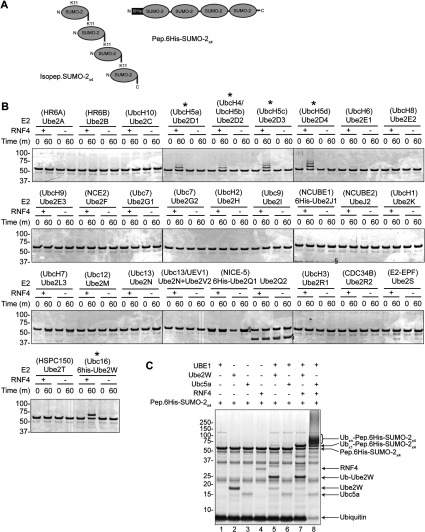
A screen for E2 enzymes that partner RNF4 in poly-SUMO ubiquitylation (**A**) Schematic representations of the native Lys^11^ isopeptide bond-linked poly-SUMO-2 construct (Isopep.SUMO-2_×4_, left-hand panel) and linear peptide bond-linked His_6_-tag poly-SUMO-2 construct (Pep.6His-SUMO-2_×4_, right-hand panel) used as substrates in the present study. (**B**) An *in vitro* screen of 29 different E2 ubiquitin-conjugation enzymes (http://www.ubiquigent.com) was undertaken using recombinant ubiquitin, UBE1 and RNF4, with Pep.6His-SUMO-2_×4_ as the substrate (see the Experimental section for details). Reactions were incubated for 60  min and products are shown on Coomassie Blue-stained SDS/PAGE. Only five E2 enzymes showed activity in this assay (as indicated by the asterisks). The § symbol marks the position of E2 enzymes. Anti-ubiquitin antibody Western blots of these reactions can be seen in Supplementary Figure S1(C) (at http://www.biochemj.org/bj/453/bj4530137add.htm). Coomassie Blue-stained SDS/PAGE of *in vitro* ubiquitylation reactions using the indicated combinations of constituents and 60-min incubation (see the Experimental section for details). Ub, ubiquitin.

**Figure 2:**
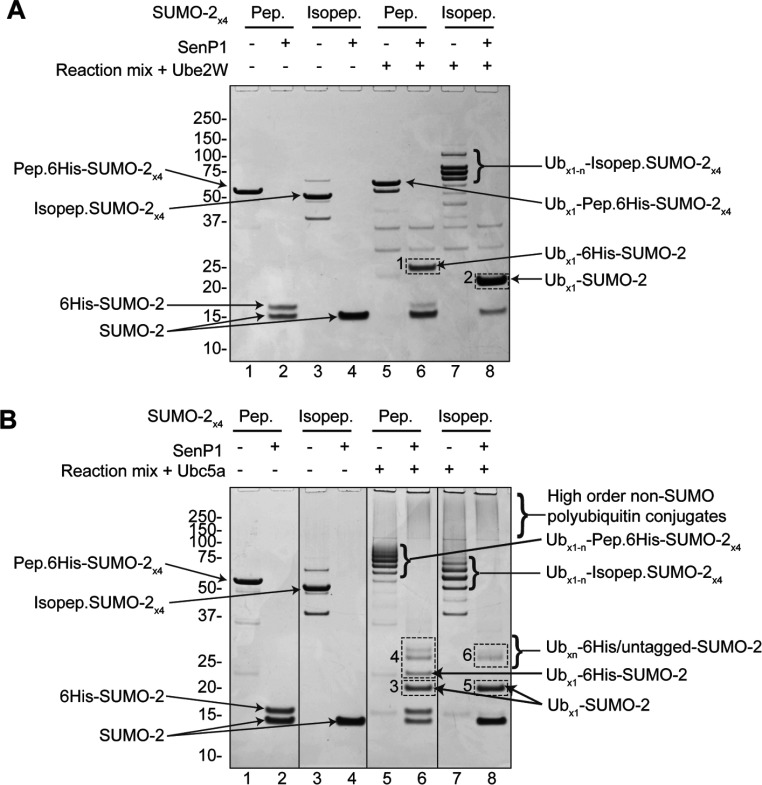
Ube2W conjugates multiple copies of ubiquitin to isopeptide-linked SUMO-2 polymers, but only mono-ubiquitinates peptide-linked SUMO-2 polymers Coomassie Blue-stained SDS/PAGE showing the reaction products of two sets of *in vitro* ubiquitylation assays using either Ube2W (**A**) or UbcH5a (**B**). Reactions were incubated for 30 min and following completion of assays, half of the reaction volumes were treated with the SUMO protease SENP1 to depolymerize all SUMO-2. Sites of lysine residue ubiquitylation, as identified from the gel areas (broken-lined boxes marked 1–6), are summarized in Supplementary Table S1 (at http://www.biochemj.org/bj/453/bj4530137add.htm). Pep (peptide) and Isopep (isopeptide) refer to the two different poly-SUMO-2 constructs shown in Figure 1(A). Ub, ubiquitin.

To determine which E2 enzymes function with RNF4, a screen of 29 E2 enzymes was undertaken to determine which catalysed the RNF4-dependent ubiquitylation of poly-SUMO-2 chains *in vitro*. A single polypeptide chain containing four SUMO moieties linked via C-to-N-terminal peptide bonds (Pep.6His-SUMO-2_×4_) (Figure 1A, right-hand side) was used as a substrate in the assay, which can be bound by RNF4 in a manner similar to the native isopeptide bond-linked poly-SUMO chain (Isopep.6His-SUMO-2_×4_) (Figure 1A, left-hand side). The screen showed that, along with the Ubc4/Ubc5 family, Ube2W was the only other conjugating enzyme to show RNF4-dependent activity (Figure 1B) [anti-ubiquitin antibody Western blots of these reactions can be seen in Supplementary Figure S1(C) at http://www.biochemj.org/bj/453/bj4530137add.htm]. Interestingly, the products of the reactions containing Ube2W and Ubc4/5 differ in that Ube2W appears only to mono-ubiquitylate the peptide-linked poly-SUMO-2 construct, whereas UbcH5a produced multi-ubiquitylated forms (Figure 1C, compare lanes 7 and 8). Reaction mixtures containing the native isopeptide-linked construct Isopep.SUMO-2_×4_ (Figure 1A), show that Ube2W attaches up to four copies of ubiquitin to this form (Figure 2A, compare lanes 5 and 7). To reduce the complexity of these reactions, the SUMO polymers were hydrolysed into monomers by treatment with the SUMO-specific protease SENP1 (sentrin-specific protease 1). Because His_6_-tagged SUMO-2 has a greater mass than the untagged form, depolymerizaton of Pep.6His-SUMO-2_×4_ produces two discrete species (Figure 2A, lane 2). Interestingly, it can be seen that it is the N-terminal copy of SUMO containing the His_6_ tag, that accepts the ubiquitin in these reactions (Figure 2B, compare lanes 2 and 6). It is also apparent that each SUMO moiety in the isopeptide-linked polymer is mono-ubiquitylated, as SENP1 digestion yielded only a single ubiquitin-SUMO-2 species (Figure 2B, lane 8). The strict 1:1 stoichiometry of ubiquitylation of SUMO by Ube2W is in contrast with UbcH5a, which produces multiply modified forms of SUMO in identical assays (Figure 2B).

### Ube2W catalyses the conjugation of ubiquitin on to protein N-termini

To identify sites of RNF4-dependent ubiquitin attachment to poly-SUMO-2 in reactions containing Ube2W and UbcH5a, MS was employed. Ubiquitylated SUMO-2 species were excised from gel slices shown in Figures 2(B) and 2(C), digested with trypsin and the resultant peptides analysed by MS (see the Experimental section for details). Supplementary Table S1 (at http://www.biochemj.org/bj/453/bj4530137add.htm) lists the sites of lysine residue ubiquitylation identified. Consistent with similar experiments [2], UbcH5a was found to catalyse ubiquitylation of SUMO-2 at Lys^11^ and Lys^32^, as well as to create ubiquitin polymers via Lys^6^, Lys^11^, Lys^48^ and Lys^63^. Unexpectedly, no sites of lysine residue ubiquitylation were detected in equivalent reactions containing Ube2W (Supplementary Table S1).

**Figure 3:**
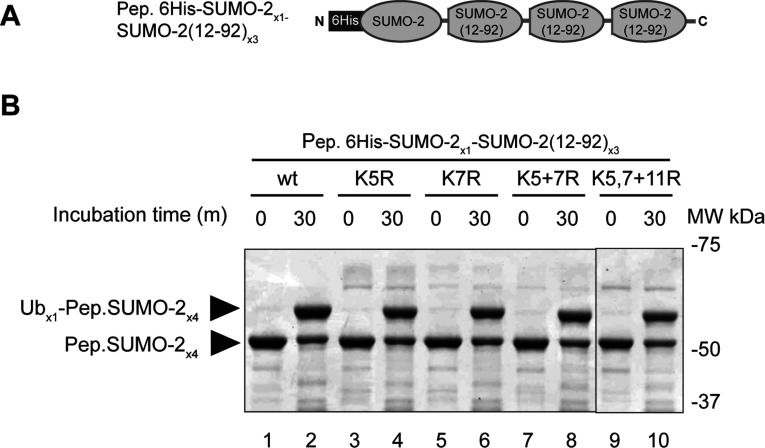
Mutations to Lys^5^, Lys^7^ and Lys^11^ of the N-terminal SUMO-2 do not affect ubiquitylation by Ube2W and RNF4 (**A**) Schematic presentation of isopeptide linked-poly-SUMO-2 with the first SUMO unit being full length, and the remaining three units lacking 11 N-terminal amino acids [Pep.6His-SUMO-2_×1_–SUMO-2-(12–92)_×3_]. This was used to create lysine residue mutants for biochemical analysis. (**B**) Four mutants of the first SUMO-2 were created, and the constructs analysed by *in vitro* ubiquitylation. Reactions were incubated for 30 min and products were visualized by Coomassie Blue-stained SDS/PAGE. MW, molecular mass; Ub, ubiquitin; wt, wild-type.

One difference between the two poly-SUMO constructs is the presence of four conformationally flexible N-terminal domains in Isopep.SUMO-2_×4_ [13] compared with only one in Pep.6His-SUMO-2_×4_ (Figure 1A). We reasoned that Ube2W was only targeting a lysine residue in a SUMO-2 N-terminus if it is not structurally constrained, and that technical limitations inhibited detection of the ubiquitylated peptide. As such, a mutagenesis study was undertaken using a new peptide-linked poly-SUMO-2 within which only the N-terminal SUMO-2 copy contained an intact N-terminus, with the remaining three copies comprising residues 12–92 (Figure 3A). Four mutants were created representing various combinations of mutations to N-terminal Lys^5^, Lys^7^ and Lys^11^, with one mutant lacking all three. Surprisingly, all constructs were efficiently mono-ubiquitylated by Ube2W in the presence of RNF4 (Figure 3B).

**Figure 4:**
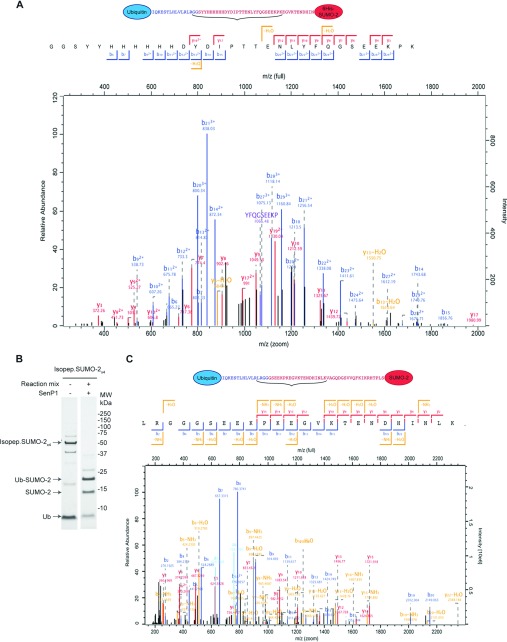
Ube2W conjugates ubiquitin to SUMO-2 protein in N-termini (**A**) Re-analysis of the MS data derived from Figure 2 revealed the presence of the peptide characteristic of N-terminally ubiquitylated Pep.6His-SUMO-2_×4_. It is noteworthy that this peptide was only detected in slice 1 of Figure 2. The N-terminal methionine residue of the 6His-poly-SUMO-2 construct is not present. (**B**) Coomassie Blue-stained SDS/PAGE analysis of *in vitro* ubiquitylation reactions using Isopep.SUMO-2_×4_ as substrate. (**C**) Partial tryptic digestion was used to identify a peptide characteristic of N-terminally ubiquitylated SUMO-2 molecules. The reaction mixture contained Ube2W and RNF4, and SENP1 was used to depolymerize the SUMO construct. MW, molecular mass.

It has been known for some time that ubiquitin can also target protein N-terminal α-amino groups [14], although few substrates have been described and the identities of the enzymes involved are largely unknown. We thus considered the possibility that SUMO was not being ubiquitylated at a lysine residue ϵ-amino group, but at the N-terminal α-amino group. Re-analysis of the MS data for slices 1–6 of Figure 2 did indeed reveal a peptide consistent with ubiquitylation at the 6His-SUMO-2 N-terminus in the peptide-linked construct (Figure 4A). This peptide was detected only in the reaction containing Ube2W and not in the equivalent reaction using UbcH5a, indicating this to be a modification that UbcH5a is incapable of creating in detectable amounts *in vitro*. Unfortunately, the ubiquitin-SUMO-2 diagnostic peptide was not found for the isopeptide-linked SUMO-2 form (Figure 2A, slice 2). Inspection of the sequence shows that if ubiquitylated at the N-terminus, trypsin cleavage would yield a short hydrophilic peptide, GGGSEEKPK, that may not bind strongly to the reverse-phase HPLC column in line with the mass spectrometer. To resolve this, a partial tryptic digestion approach in combination with altered HPLC peptide fractionation (see the Experimental section) facilitated the detection of a peptide indicative of N-terminal ubiquitylation of SUMO-2 (Figures 4B and 4C). In addition to SUMO-2 N-terminal conjugation, we found that Ube2W ubiquitylates its own N-terminus in the absence of RNF4 (Supplementary Figures 2A and 2B at http://www.biochemj.org/bj/453/bj4530137add.htm), although RNF4 does modestly accelerate the process (Supplementary Figure 2A). The N-terminal ubiquitylation of RNF4 was also detectable in these assays (Supplementary Figures 2A and 2C).

### N-terminally ubiquitylated poly-SUMO-2 is a substrate for RNF4-dependent Ubc13–UEV1-mediated poly-ubiquitylation

**Figure 5:**
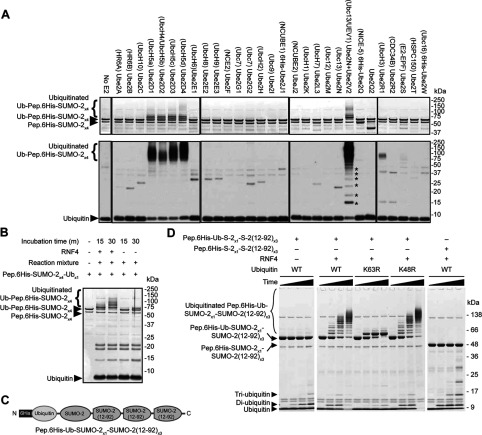
The Ubc13–Ube2V heterodimer catalyses RNF4-dependent ubiquitylation of N-terminally mono-ubiquitylated poly-SUMO-2 (**A**) Screen for E2 enzymes that recognize N-terminally mono-ubiquitylated poly-SUMO-2 as a substrate. The indicated E2 enzymes were incubated for 60 min together with ubiquitin, UBE1, Ube2W and RNF4, with mono-ubiquitylated Pep.6his-SUMO-2_×4_ as a substrate (see Experimental section). The upper panels show the Coomassie Blue-stained gel images, and the lower panels show anti-ubiquitin antibody Western blots. The asterisks indicate the position of unanchored ubiquitin chains. (**B**) *In vitro* ubiquitylation reactions using purified mono-ubiquitylated Pep.6His-SUMO-2_×4_ as a substrate and Ubc13–Ube2V as the E2 conjugating enzyme. Dependence on RNF4 and incubation time is shown. (**C**) Schematic presentation of the recombinantly expressed ubiquitin–poly-SUMO-2 construct used in (**D**). (**D**) Ubc13, Ube2V2, UBE1 and ubiquitin [either wild-type (WT) or mutant] were incubated with RNF4 and substrate [either Pep.6His-Ub-SUMO-2_×1_–SUMO-2-(12–92)_×3_ (see Figure 3A) or Pep.6His-SUMO-2_×1_–SUMO-2-(12–92)_×3_] as indicated. Reaction time points were taken at 0, 10, 30 and 100 min. The reactions were analysed by SDS/PAGE, followed by Coomassie Blue staining.

As Ube2W only mono-ubiquitylated SUMO-2 N-termini in the assays in the present study with very little evidence for lysine residue ubiquitylation, we considered the possibility that N-terminally ubiquitylated SUMO polymers may act as a substrate for further rounds of ubiquitylation by other enzymes. To test this hypothesis, the ubiquitin E2 enzyme screen was repeated, this time using purified N-terminally ubiquitylated SUMO-2 as the substrate (Figure 5A). As before the Ubc4/5 E2s ubiquitylated this substrate, but now the Ube2N–Ube2V1 (Ubc13—UEV1) heterodimeric E2 complex could ubiquitylate where previously it did not (compare Figure 5A with Figure 1B), in a manner dependent on RNF4 (Figure 5B). A bacterially expressed single polypeptide construct of the form 6His-Ub-SUMO-2_×4_ (Figure 5C) was also an efficient substrate and mutants of ubiquitin revealed the chains to be exclusively Lys^63^linked (Figure 5D).

### Ube2W N-terminally mono-ubiquitinates the ubiquitin E3 ligase CHIP

**Figure 6:**
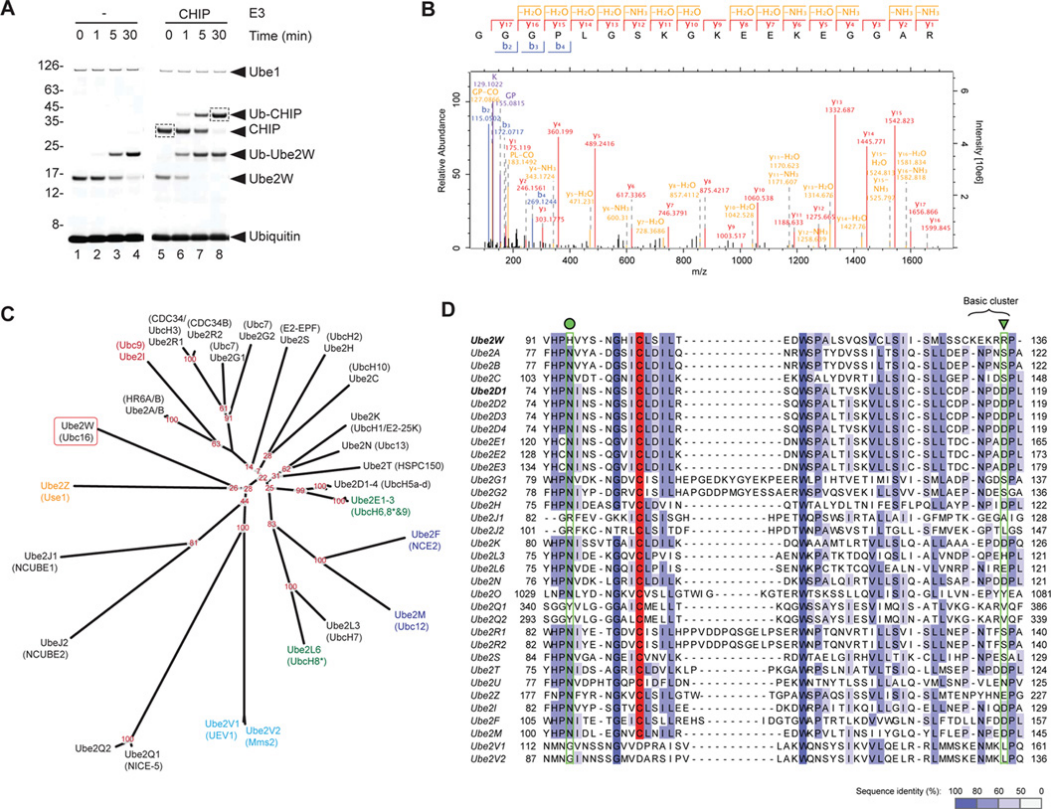
The general function of Ube2W is as a protein N-terminal ubiquitin-conjugating enzyme (**A**) Coomassie Blue-stained SDS/PAGE analysis of the products of *in vitro* ubiquitylation assays containing Ube2W and CHIP (as indicated by the broken-lined boxes). (**B**) MS/MS spectrum of the peptide diagnostic of N-terminal ubiquitylation of CHIP. (**C**) Consensus phylogenetic tree depicting the protein sequence-derived relationship between 32 proteins with sequence homology to ubiquitin E2-conjugating enzymes. The red numbers indicate consensus tree bootstrap percentages for nodes. The enzymes are colour-coded by known modifier specificity: black, ubiquitin/putative ubiquitin; red, SUMO; blue, NEDD8 (neural precursor cell expressed, developmentally down-regulated 8); green, ISG15 (ubiquitin-like protein ISG15); orange, FAT10 (also known as UBD; ubiquitin D); cyan, catalytically inactive. *Ube2E2 and Ube2L6 have both been known as UbcH8. Phylogenies created by PHYLIP (http://cmgm.stanford.edu/phylip/). (**D**) Sequence alignment of domains surrounding the catalytic cysteine residues (red) of human E2 enzymes. The position of Asn^77^ of Ubch5A (Ube2D1) is marked with a green circle, and the UbcH5a Asp^117^ is marked with a green triangle. The alignment was created in JalView (http://www.jalview.org/) [29].

CHIP is a U-box E3 ubiquitin ligase known to interact productively with many E2 enzymes. Indeed, it has been shown that Ube2W efficiently mono-ubiquitylates CHIP *in vitro* [15,16]. To determine whether Ube2W generally functions as a protein N-terminus-conjugating enzyme, we synthesized Ube2W-dependent mono-ubiquitinated CHIP (Figure 6A) and determined the sites of conjugation using MS. No sites of lysine residue ubiquitylation were found, but the peptide indicative of N-terminal ubiquitylation was clearly detected (Figure 6B). This strongly suggests that the general function of Ube2W is to catalyse the synthesis of peptide linkages between the C-terminus of ubiquitin and the N-terminus of substrates.

## DISCUSSION

We have shown that the ubiquitin-conjugating enzyme Ube2W (Ubc16) functions together with RNF4 to attach a single copy of ubiquitin to the N-terminal α-amino group of SUMO molecules in poly-SUMO chains. The modification of protein N-termini by ubiquitin could regulate substrate stability or function in many ways, such as altering the N-degron [17], targeting for the UFD (ubiquitin fusion degradation) pathway [18] or triggering further modifications such as Lys^63^ poly-ubiquitylation as demonstrated in the present study.

Protein N-terminal ubiquitylation has been described previously although no specific E2s have been identified [14,19,20]. A phylogenetic analysis (Figure 6C) shows Ube2W as not being particularly closely related to other E2s, with its nearest relatives including the inactive Ube2V1 and Ube2V2, and the largely uncharacterized Ube2J and Ube2Q subfamilies.

These are significantly different in sequence from well-characterized ubiquitin-conjugating enzymes (Figure 6D). Taking into consideration these bioinformatic analyses and the fact that the promiscuous E2 UbcH5a was unable to N-terminally ubiquitylate SUMO in the present assays, we suggest that Ube2W uniquely functions *in vivo* as a ubiquitin-conjugating enzyme specific for protein N-termini. We cannot exclude the possibility that Ube2W specificity could be influenced by other factors, neither do we contend that Ube2W is solely responsible for all cellular N-terminal ubiquitylation. Indeed, the E3 ligase LUBAC (linear ubiquitin chain assembly complex) can synthesize linear poly-ubiqitin chains by partnering with many E2s including E2-25K, UbcH7, Ube2B and the UbcH4/5 family [21].

A key outcome of the present study is to highlight the importance of considering N-terminal ubiquitylation. Ube2W has been shown to be responsible for the mono-ubiquitylation of BRCA1 (breast cancer early-onset 1) [22], Fanconi's anaemia proteins FANCL (Fanconi's anaemia, complementation group L) and FANCD2 (Fanconi's anaemia, complementation group D2) [23,24] and CHIP [15,16]. Only one ubiquitylation site was described, which in the case of CHIP was shown by MS analysis to be Lys^2^, the mutation of which did not inhibit modification [16]. In the present study, in an analysis of CHIP mono-ubiquitylation by Ube2W, we found no evidence of lysine residue modification, with a very clearly identified peptide diagnostic of N-terminal ubiquitylation. In general, we suspect that in these cases of mono-ubiquitylation catalysed by Ube2W described above, lysine residue modification is at trace levels, and that N-terminal modification was not detected simply because it was not specifically sought.

The present study raises the question of how Ube2W is able to discriminate between the ϵ-amino group of lysine residue side chains and N-terminal α-amino groups. One key difference between them is their ionization state. Although the p*K*_a_ of lysine residue side chains is 10.5±1.1, the p*K*_a_ of N-terminal amino groups is 7.7±0.5 [25]. Thus, at physiological pH, only a very small proportion of ϵ-amino groups will be uncharged. Importantly, Ube2W lacks a well-conserved acidic residue close to the active site, that in UbcH5a (Asp^117^) is thought to be important for placement and deprotonation of the ϵ-amino group of an incoming substrate lysine residue [26]. The equivalent region in Ube2W is largely basic in character (Figure 2d), which would likely exclude a positively charged lysine side chain from approaching the active site, but would accommodate a neutral N-terminal amino group. This basic cluster might also help orient the incoming protein N-terminus by interacting with the partial negative charge on the oxygen of the first peptide bond carbonyl. Another key residue of UbcH5a is Asn^77^ (Figure 6B), which has been proposed to stabilize the oxyanion of the tetrahedral transition state [27], and to play a structural role by hydrogen bonding to the peptide backbone of the active-site loop [28]. This asparagine residue is conserved in most other active E2s, whereas in Ube2W the equivalent residue is histidine (Figure 6B). However, provided that this histidine residue is protonated (p*K*_a_ 6.6±1.0), it should also be capable of stabilizing the transition state oxyanion and of stabilizing the active-site loop. This idea is supported by pH titration analysis which showed that in contrast with UbcH5a, Ube2W displays a striking pH-dependence being largely inactive above pH 8.0 (Supplementary Figure S3 at http://www.biochemj.org/bj/453/bj4530137add.htm). It is not clear how this might favour N-terminal modification, but it is possible that the carbonyl oxygen of the first peptide bond could disfavour formation of the oxyanion and this might be facilitated by the strong positive charge on the histidine residue.

Another important question is which structural features of substrates regulate N-terminal ubiquitylation by Ube2W? In the experiments described in the present study, a variety of N-terminal sequences, including those with long N-terminal affinity tags, could function as ubiquitin acceptors (for example, see Figure 2). However, it is intriguing to note that proteins N-terminally ubiquitinated by Ube2W that still contain an N-terminus (from ubiquitin) were not substrates for further rounds of N-terminal conjugation (Figures 1–4). This is true even if the same affinity tag sequence is present on ubiquitin that was shown previously to be N-terminally modified in the context of the Pep.6His-SUMO-2_×4_ substrate (compare Figure 1 with Figure 5). Although further experimentation is necessary to investigate these issues, we suspect that a number of features will contribute to protein N-terminal ubiquitylation efficiency, including substrate N-terminal primary and secondary structures, post-translational modifications (such as N-terminal acetylation) and proximity of the N-terminus to the active site of the E2 ubiquitin–E3 substrate complex.

## Online data

Supplementary data
