# Supplementary material for: Ube2W conjugates ubiquitin to α-amino groups of protein N-termini
Source: Biochem J. 2013 Jun 13;453(Pt 1):137–45. doi: 10.1042/BJ20130244 (PMC3778709; doi:10.1042/BJ20130244)
Supplement: Supplementary data [file bj4530137add.pdf]

BJ Signal

Biochem. J. (2013) **453**, 137–145 (Printed in Great Britain) doi:10.1042/BJ20130244

online data

SUPPLEMENTARY ONLINE DATA

Ube2W conjugates ubiquitin to α-amino groups of protein N-termini

Michael H. TATHAM, Anna PLECHANOVOVÁ, Ellis G. JAFFRAY, Helena SALMEN and Ronald T. HAY[1]

Wellcome Trust Centre for Gene Regulation and Expression, Sir James Black Centre, College of Life Sciences, University of Dundee, Dow Street, Dundee DD1 5EH, U.K.

PROTEIN SEQUENCES

Ube2W

GAMGSMASMQTTGRRVEVWFPKRLQKELLALQNDPPP-
GMTLNEKSVQNSITQWIVDMEGAPGTLYEGEKFQLLFK-
FSSRYPFDSPQVMFTGENIPVHPHVYSNGHICLSILTEDW-
SPALSVQSVCLSIISMLSSCKEKRRPPDNSFYVRTCNKNP-
KKTKWWYHDDTC

Pep.6His-SUMO-2$_{\times 4}$

MSYYHHHHHHDYDIPTTENLYFQGSEEKPKEGVKTEND-
HINLKVAGQDGSVVQFKIKRHTPLSKLMKAYCERQGLS-
MRQIRFRFDGQPINETDTPAQLEMEDEDTIDVFQQQTGG-
GSEEKPKEGVKTENDHINLKVAGQDGSVVQFKIKRHTP-
LSKLMKAYCERQGLSMRQIRFRFDGQPINETDTPAQLEM-
EDEDTIDVFQQQTGGGSEEKPKEGVKTENDHINLKVAGQ-
DGSVVQFKIKRHTPLSKLMKAYCERQGLSMRQIRFRFDG-
QPINETDTPAQLEMEDEDTIDVFQQQTGGGSEEKPKEGV-
KTENDHINLKVAGQDGSVVQFKIKRHTPLSKLMKAYCE-
RQGLSMRQIRFRFDGQPINETDTPAQLEMEDEDTIDVFQ-
QQTGG

RNF4

GAMDHVEFGSMSTRNPQRKRRGGAVNSRQTQKRTRETTS-
TPEISLEAEPIELVETVGDEIVDLTCESLEPVVVDLTHNDSV-
VIVEERRRPRRNGRRLRQDHADSCVVSSDDEELSKDKDV-
YVTTHTPRSTKDEGTTGLRPSGTVSCPICMDGYSEIVQNG-
RLIVSTECGHVFCSQCLRDSLKNANTCPTCRKKINHKRYH-
PIYI

SUMO-2 (used to create Isopep.SUMO-2$_{\times 4}$)

GSEEKPKEGVKTENDHINLKVAGQDGSVVQFKIKRHTPLS-
KLMKAYCERQGLSMRQIRFRFDGQPINETDTPAQLEMEDE-
DTIDVFQQQTGG

6His-UBE1

MSYYHHHHHHDYDIPTTENLYFQGAMGSSSSPLSKKRRVS-
GPDPKPGSNCSPAQSVLSEVPSVPTNGMAKNGSEADIDEGL-
YSRQLYVLGHEAMKRLQTSSVLVSGLRGLGVEIAKNIILGG-
VKAVTLHDQGTAQWADLSSQFYLREEDIGKNRAEVSQPRL-
AELNSYVPVTAYTGPLVEDFLSGFQVVVLTNTPLEDQLRVG-
EICHNRGIKLVVADTRGLFGQLFCDFGEEMILTDSNGEQPLS-
AMVSMVTKDNPGVVTCLDEARHGFESGDFVSFSEVQGMV-
ELNGNQPMEIKVLGPYTFSICDTSNFSDYIRGGIVSQVKVPK-
KISFKSLVASLAEPDFVVTDFAKFSRPAQLHIGFQALHQFCA-
QHGRPPRPRNEEDAAELVALAQAVNARALPAVQQNNLDED-
LIRKLAYVAAGDLAPINAFIGGLAAQEVMKACSGKFMPIMQ-
WLYFDALECLPEDKEVLTEDKCLQRQNRYDGQVAVFGSD-
LQEKLGKQKYFLVGAGAIGCELLKNFAMIGLGCGEGGEI-
IVTDMDTIEKSNLNRQFLFRPWDVTKLKSDTAAAAVRQM-
NPHIRVTSHQNRVGPDTERIYDDDFFQNLDGVANALDNV-
DARMYMDRRCVYYRKPLLESGTLGTKGNVQVVIPFLTE-
SYSSSQDPPEKSIPICTLKNFPNAIEHTLQWARDEFEGLFK-
QPAENVNQYLTDPKFVERTLRLAGTQPLEVLEAVQRSLVL-
QRPQTWADCVTWACHHWHTQYSNNIRQLLHNFPPDQLT-
SSGAPFWSGPKRCPHPLTFDVNNPLHLDYVMAAANLFA-
QTYGLTGSQDRAAVATFLQSVQVPEFTPKSGVKIHVSDQE-
LQSANASVDDSRLEELKATLPSPDKLPGFKMYPIDFEKDD-
DSNFHMDFIVAASNLRAENYDIPSADRHKSKLIAGKIIPAI-
ATTTAAVVGLVCLELYKVVQGHRQLDSYKNGFLNLALPF-
FGFSEPLAAPRHQYYNQEWTLWDRFEVQGLQPNGEEMT-
LKQFLDYFKTEHKLEITMLSQGVSMLYSFFMPAAKLKER-
LDQPMTEIVSRVSKRKLGRHVRALVLELCCNDESGEDVE-
VPYVRYTIR

Ubiquitin

MQIFVKTLTGKTITLEVEPSDTIENVKAKIQDKEGIPPDQ-
QRLIFAGKQLEDGRTLSDYNIQKESTLHLVLRLRGG

UbcH5a

GAMALKRIQKELSDLQRDPPAHCSAGPVGDDLFHWQAT-
IMGPPDSAYQGGVFFLTVHFPTDYPFKPPKIAFTTKIYHPN-
INSNGSICLDILRSQWSPALTVSKVLLSICSLLCDPNPDDP-
LVPDIAQIYKSDKEKYNRHAREWTQKYAM

Pep.6His-SUMO-2$_{\times 1}$–SUMO-2-(12–92)$_{\times 3}$ (also used for N-terminal mutational analysis at the underlined residues)

MSYYHHHHHHDYDIPTTENLYFQGSEEKPKEGVKTEND-
HINLKVAGQDGSVVQFKIKRHTPLSKLMKAYCERQGLS-
MRQIRFRFDGQPINETDTPAQLEMEDEDTIDVFQQQTGGG-
STENDHINLKVAGQDGSVVQFKIKRHTPLSKLMKAYCER-
QGLSMRQIRFRFDGQPINETDTPAQLEMEDEDTIDVFQQQ-
TGGGSTENDHINLKVAGQDGSVVQFKIKRHTPLSKLMKA-
YCERQGLSMRQIRFRFDGQPINETDTPAQLEMEDEDTIDV-
FQQQTGGGSTENDHINLKVAGQDGSVVQFKIKRHTPLSK-
LMKAYCERQGLSMRQIRFRFDGQPINETDTPAQLEMEDE-
DTIDVFQQQTGG

Pep.6His-Ub-SUMO-2$_{\times 1}$–SUMO-2-(12–92)$_{\times 3}$

MSYYHHHHHHDYDIPTTENLYFQGAMGMQIFVKTLTGK-
TITLEVEPSDTIENVKAKIQDKEGIPPDQQRLIFAGKQLED-
GRTLSDYNIQKESTLHLVLRLRGGGSEEKPKEGVKTEND-
HINLKVAGQDGSVVQFKIKRHTPLSKLMKAYCERQGLSM-
RQIRFRFDGQPINETDTPAQLEMEDEDTIDVFQQQTGGG-

[1] To whom correspondence should be addressed (email R.T.Hay@dundee.ac.uk).

© 2013 The Author(s)
The author(s) has paid for this article to be freely available under the terms of the Creative Commons Attribution Licence (CC-BY) (http://creativecommons.org/licenses/by/3.0/) which permits unrestricted use, distribution and reproduction in any medium, provided the original work is properly cited.

Biochemical Journal www.biochemj.org

STENDHINLKVAGQDGSVVQFKIKRHTPLSKLMKAYCERQ-
GLSMRQIRFRFDGQPINETDTPAQLEMEDEDTIDVFQQQTG-
GGSTENDHINLKVAGQDGSVVQFKIKRHTPLSKLMKAYCE-
RQGLSMRQIRFRFDGQPINETDTPAQLEMEDEDTIDVFQQQ-
TGGGSTENDHINLKVAGQDGSVVQFKIKRHTPLSKLMKAY-
CERQGLSMRQIRFRFDGQPINETDTPAQLEMEDEDTIDVFQ-
QQTGG

CHIP

GPLGSKGKEEKEGGARLGAGGGSPEKSPSAQELKEQGNRL-
FVGRKYPEAAACYGRAITRNPLVAVYYTNRALCYLKMQQH-
EQALADCRRALELDGQSVKAHFFLGQCQLEMESYDEAIAN-
LQRAYSLAKEQRLNFGDDIPSALRIAKKKRWNSIEERRIHQ-
ESELHSYLSRLIAAERERELEECQRNHEGDEDDSHVRAQQ-
ACIEAKHDKYMADMDELFSQVDEKRKKRDIPDYLCGKIS-
FELMREPCITPSGITYDRKDIEEHLQRVGHFDPVTRSPLTQ-
EQLIPNLAMKEVIDAFISENGWVEDY

Peptide-linked poly-SUMO proteins

Peptide-linked poly-SUMO constructs were cloned and the proteins were expressed and purified essentially as described previously [1]. Previous work showed that Pep.6His-SUMO-$2_{\times 4}$ (Figure 1A of the main text) behaved in a similar manner to the native isopeptide bond-linked polymer (Pep.SUMO-$2_{\times 4}$) in RNF4-dependent *in vitro* ubiquitin conjugation reactions [1]. It was used in initial screening studies because large quantities can be readily obtained by standard procedures in comparison with enzyme-synthesized isopeptide polymers, and it was assumed to be a functional mimetic of the native SUMO polymer.

Table S1 No sites of SUMO or ubiquitin lysine ubiquitination were detected in reactions containing Ube2W

Sites of lysine residue ubiquitylation detected by MS analysis of gel sections shown in Supplementary Figures S2(B) and S2(C). It is noteworthy that peptide intensities are shown as $\log_{10}$ values, and anything lower than approximately $\times 10^5$ is essentially undetectable in this system. Also, peptide intensities are indicative of the abundance of the same peptide among different slices, but not necessarily of abundance of the different peptides in the same slice.

Ubiquitylation site	Peptide intensity ($\times 10^5$)					
	Slice 1	Slice 2	Slice 3	Slice 4	Slice 5	Slice 6
SUMO-2 (Lys^{11})	–	–	7.91	7.67	7.74	6.58
SUMO-2 (Lys^{32})	–	–	7.78	7.68	7.88	7.62
Ubiquitin (Lys^{6})	–	–	–	7.07	–	7.41
Ubiquitin (Lys^{11})	–	–	–	8.68	6.81	8.65
Ubiquitin (Lys^{48})	–	–	6.85	8.54	–	8.62
Ubiquitin (Lys^{63})	–	–	6.50	8.38	–	8.34

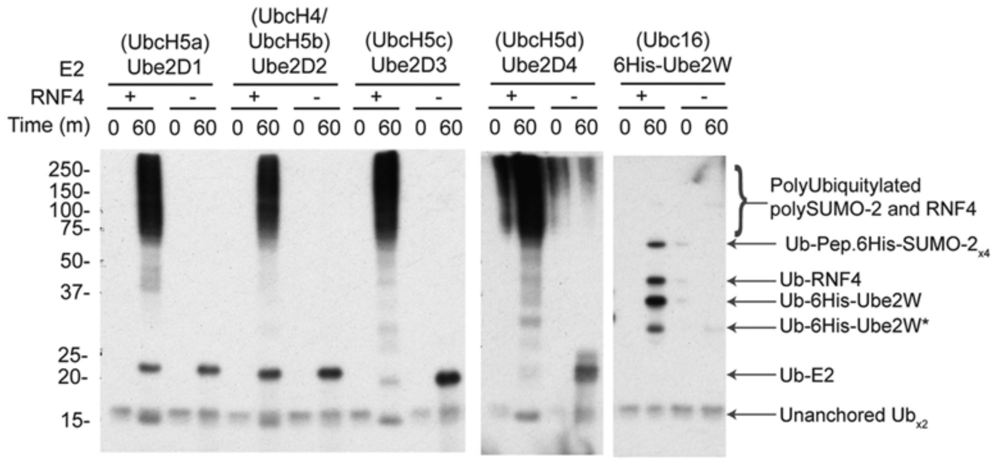

Figure S1 Anti-ubiquitin antibody Western blots of samples shown in Figure 1(B) of the main text

Anti-ubiquitin antibody Western blots showing the conjugated forms of ubiquitin detected for the samples shown in Figure 1(B) of the main text for E2 enzymes displaying RNF4-dependent poly-SUMO-2 ubiquitylation activity. Multiple species reactive to the ubiquitin antibody can be seen in the His_6-Ube2W reaction which are predicted to be mono-ubiquitinated forms of His_6-Ube2W, RNF4 and a breakdown product of His_6-Ube2W (indicated by *). It is worth noting that the polyclonal rabbit anti-ubiquitin antibody (DAKO) appears to have lower affinity for mono-ubiquitylated forms of conjugates than other forms, so disproportionately reacts to ubiquitin depending on its conjugation state. Ub, ubiquitin.

© 2013 The Author(s)
The author(s) has paid for this article to be freely available under the terms of the Creative Commons Attribution Licence (CC-BY) (http://creativecommons.org/licenses/by/3.0/) which permits unrestricted use, distribution and reproduction in any medium, provided the original work is properly cited.

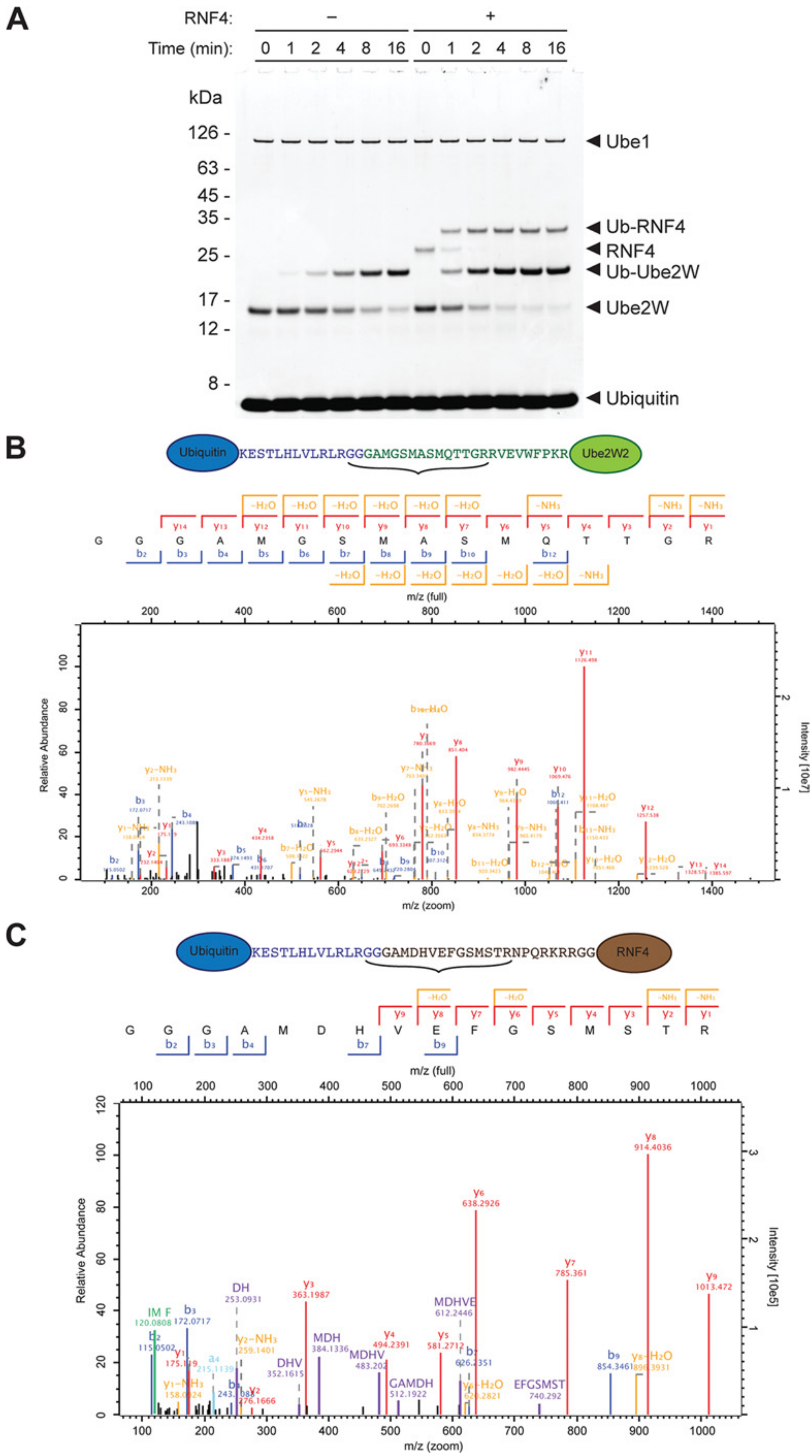

© 2013 The Author(s)
The author(s) has paid for this article to be freely available under the terms of the Creative Commons Attribution Licence (CC-BY) (http://creativecommons.org/licenses/by/3.0/) which permits unrestricted use, distribution and reproduction in any medium, provided the original work is properly cited.

Figure S2 Ube2W and RNF4 are N-terminally ubiquitinated *in vitro*

(**A**) Ube1 (0.1 μM) and ubiquitin (20 μM) were incubated at room temperature either in the presence or absence of RNF4 (0.55 μM). Samples were taken at the indicated time points and analysed by reducing SDS/PAGE, followed by staining with Coomassie Blue. (**B**) MS/MS spectrum of the Ub-Ube2W peptide detected by in-gel digestion from similar experiments. (**C**) MS/MS spectrum of the peptide indicative of N-terminally ubiquitinated RNF4 detected by in-solution digestion of the reaction products shown in Figure 3, lane 2, in the main text. Ub, ubiquitin.

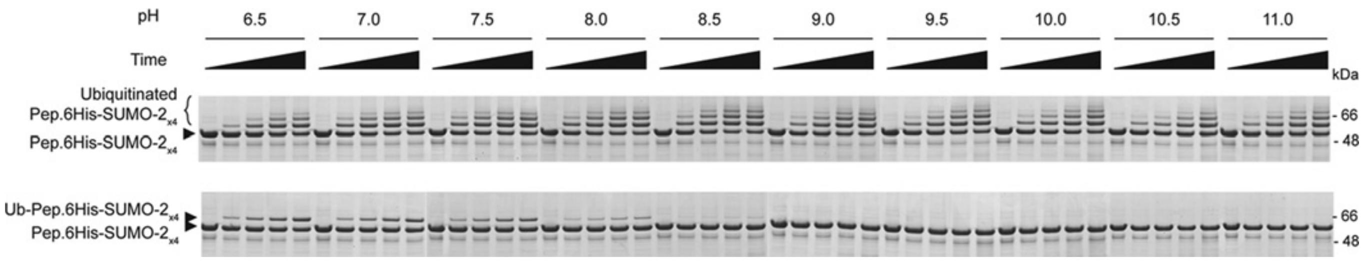

Figure S3 Ube2W has low activity at alkaline pH

Coomassie Blue-stained SDS/PAGE images of *in vitro* conjugation reactions containing ubiquitin, UBE1, RNF4 and either UbcH5a (upper panels) or Ube2W (lower panels). The reactions were buffered to different pH levels and were monitored at 0, 15, 30, 60 and 120 min.

REFERENCES

1 Tatham, M. H., Geoffroy, M. C., Shen, L., Plechanovova, A., Hattersley, N., Jaffray, E. G., Palvimo, J. J. and Hay, R. T. (2008) RNF4 is a poly-SUMO-specific E3 ubiquitin ligase required for arsenic-induced PML degradation. Nat. Cell Biol. **10**, 538–546

Received 18 February 2013/2 April 2013; accepted 8 April 2013
Published as BJ Immediate Publication 8 April 2013, doi:10.1042/BJ20130244

© 2013 The Author(s)
The author(s) has paid for this article to be freely available under the terms of the Creative Commons Attribution Licence (CC-BY) (http://creativecommons.org/licenses/by/3.0/) which permits unrestricted use, distribution and reproduction in any medium, provided the original work is properly cited.
